# TGF-β1 Downregulates COX-2 Expression Leading to Decrease of PGE2 Production in Human Lung Cancer A549 Cells, Which Is Involved in Fibrotic Response to TGF-β1

**DOI:** 10.1371/journal.pone.0076346

**Published:** 2013-10-02

**Authors:** Erina Takai, Mitsutoshi Tsukimoto, Shuji Kojima

**Affiliations:** Department of Radiation Biosciences, Faculty of Pharmaceutical Sciences, Tokyo University of Science, Noda-shi, Chiba, Japan; Rajiv Gandhi Centre for Biotechnology, India

## Abstract

Transforming growth factor-ß1 (TGF-β1) is a multifunctional cytokine that is involved in various pathophysiological processes, including cancer progression and fibrotic disorders. Here, we show that treatment with TGF-β1 (5 ng/mL) induced downregulation of cyclooxygenase-2 (COX-2), leading to reduced synthesis of prostaglandin E2 (PGE2), in human lung cancer A549 cells. Treatment of cells with specific inhibitors of COX-2 or PGE2 receptor resulted in growth inhibition, indicating that the COX-2/PGE2 pathway contributes to proliferation in an autocrine manner. TGF-β1 treatment induced growth inhibition, which was attenuated by exogenous PGE2. TGF-β1 is also a potent inducer of epithelial mesenchymal transition (EMT), a phenotype change in which epithelial cells differentiate into fibroblastoid cells. Supplementation with PGE2 or PGE2 receptor EP4 agonist PGE1-alcohol, as compared with EP1/3 agonist sulprostone, inhibited TGF-β1-induced expression of fibronectin and collagen I (extracellular matrix components). Exogenous PGE2 or PGE2 receptor agonists also suppressed actin remodeling induced by TGF-β1. These results suggest that PGE2 has an anti-fibrotic effect. We conclude that TGF-β1-induced downregulation of COX-2/PGE2 signaling is involved in facilitation of fibrotic EMT response in A549 cells.

## Introduction

Transforming growth factor-ß1 (TGF-β1) was originally discovered as a secreted protein that induces transformation and growth of normal fibroblasts [1], and it is now well established to be involved in various fibrotic disorders, such as pulmonary and hepatic fibrosis [2-4]. Indeed, TGF-β1 is a multifunctional cytokine that regulates various physiological processes, including cell growth, differentiation, and tumorigenesis. It is secreted into tumor microenvironments from many cancer cells and acts as a tumor promoter by inducing angiogenesis, immune-escape and metastasis [5-7]. Lung fibrosis, such as idiopathic pulmonary fibrosis (IPF), is well known to be associated with increased risk of lung cancer. On the other hand, it has also been proposed that fibrosis in lung tumor is a secondary phenomenon rather than a precursor of cancer, and it was demonstrated that the degree of fibrosis is correlated with cancer progression and prognosis [8,9]. However, the mechanisms of development of fibrosis in lung tumor are not well understood.

In epithelial cells, TGF-β1 induces a phenotype change called epithelial mesenchymal transition (EMT), which is the process through which epithelial cells differentiate into fibroblast-like mesenchymal cells. EMT is a normal physiological process essential for proper embryogenesis and tissue morphogenesis. On the other hand, EMT is also involved in wound repair and cancer progression in adult tissues [10,11]. Though a contribution of EMT-derived fibroblast-like cells to fibrosis has been suggested [12,13], the signaling mechanisms underlying TGF-β1-induced biological events in cancer cells are not fully understood.

Cyclooxygenase (COX) is the rate-limiting enzyme in prostanoid synthesis. While COX-1 is constitutively expressed, COX-2 is inducible, and is well known to be involved in inflammation. Expression of COX-2 is elevated in many tumor tissues, including lung cancer [14,15]. Prostaglandin E2 (PGE2), which is the predominant prostaglandin, exerts its biological effects via G protein-coupled receptors (i.e., EP1-EP4) [16]. It has been reported that PGE2 is involved in tumor growth, immunosuppression, or angiogenesis [17-20]. It has also been reported that COX-2 is overexpressed in many lung cancers, and PGE2 is involved in proliferation, resistance to apoptosis and induction of T-regulatory cells [21-24]. In non-small cell lung cancer (NSCLC), overexpression or activating mutation of epidermal growth factor receptor (EGFR) leads to aberrant proliferation or migration. Hence, EGFR is established as a molecular marker of NSCLC, and is widely used for prediction of prognosis or for treatment choice. COX-2, as well as EGFR, is a possible molecular marker of NSCLC [14].

We have recently reported that treatment of human NSCLC A549 cells with TGF-β1 induces EMT leading to enhancement of cell migration [25]. In the present study, to reveal the mechanism of TGF-β1-induced lung cancer progression, we investigated the effect of TGF-β1 on expression of COX-2 in A549 cells. Previously, it has been reported that TGF-β1 induces COX-2 expression during EMT in mammary epithelial cells [26]. Contrary to the case in mammary epithelial cells, we found for the first time that TGF-β1 downregulates COX-2 in human NSCLC A549 cells. We show here that this effect is related to growth inhibition and facilitation of fibrotic EMT response, suggesting that COX-2/PGE2 signaling is important for the control of cellular processes in A549 cells.

## Materials and Methods

### Reagents and antibodies

DMEM, human recombinant TGF-β1, SB431542, cycloheximide and methyl acetate were purchased from Wako Pure Chemical (Osaka, Japan). FBS was purchased from Biowest (Nuaillé, France). AH6809, L798106, L161982, sulprostone, butaprost and PGE1-alcohol were purchased from Cayman Chemical (Ann Arbor, MI, USA). Prostaglandin E2, actinomycin D and anti-N-cadherin antibody were purchased from Sigma-Aldrich (St Louis, MO, USA). Specific inhibitor of Smad3 (SIS3) and NS-398 were purchased from Merck (Darmstadt, Germany). Rhodamine phalloidin was purchased from Cytoskeleton, Inc. (Denver, CO, USA). Rabbit polyclonal anti-COX-2 antibody was purchased from Cell Signaling Technology, Inc. (Danvers, MA, USA). Rabbit polyclonal anti-high mobility group box 1 (HMGB1) antibody was purchased from Abcam (Cambridge, UK). Rabbit polyclonal anti-COX-1 antibody and mouse monoclonal anti-actin antibody were purchased from Santa Cruz Biotechnology, Inc. (Santa Cruz, CA, USA). Mouse monoclonal anti-E-cadherin antibody (Clone 36B5) was purchased from Thermo, Fisher Scientific (Waltham, MA, USA). Mouse monoclonal anti-fibronectin antibody was purchased from BD Biosciences (Franklin Lakes, NJ, USA).

### Cell culture

A549 human adenocarcinoma cells were obtained from American Type Culture Collection (ATCC, Manassas, VA, USA). Cells were grown in DMEM supplemented with 10% fetal bovine serum, penicillin (100 units/mL) and streptomycin (100 mg/mL) in a humidified atmosphere of 5% CO_2_ in air at 37 °C.

### Immunoblotting

Cells were lysed in PBS containing 10 mM HEPES-NaOH, pH 7.4, 1% Triton X100, 5 mM EDTA, 30 mM sodium pyrophosphate, 50 mM sodium fluoride, 1 mM sodium orthovanadate, 1.04 mM 4-(2-aminoethyl)benzenesulfonyl fluoride, 0.8 µM aprotinin, 21 µM leupeptin, 36 µM bestatin, 15 µM pepstatin A and 14 µM E-64, at 4 °C for 30 min. Lysates were centrifuged at 10,000 x g for 15 min. After removal of cellular debris, the protein content in each sample was determined using the Bio-Rad Protein Assay kit (Bio-Rad Laboratories, Hercules, CA, USA). Equal amounts of protein lysate were dissolved in 2 x sample buffer (50% glycerin, 2% SDS, 125 mM Tris, 10 mM DTT) and boiled for 10 min. Aliquots of samples containing 6 µg of protein were analyzed by means of 7.5-15% SDS-PAGE and bands were transferred onto a PVDF membrane. The blots were blocked overnight in TBST with 1% BSA, 5% skim milk at 4 °C, then incubated with rabbit COX-2 antibody (1:1000), rabbit COX-1 antibody (1:1000), rabbit HMGB1 antibody (1:1000), mouse actin antibody (1:1000), mouse E-cadherin antibody (1:1000), mouse N-cadherin antibody (1:1000) or mouse fibronectin antibody (1:1000) overnight at 4 °C. After having been washed with TBST, blots were incubated with goat HRP-conjugated anti-rabbit IgG antibody (Cell Signaling Technology, Inc.) or goat anti-mouse IgG antibody-HRP (Santa Cruz Biotechnology) for 90 min at room temperature. The blots were again washed with TBST, and specific proteins were visualized by using ECL Western blotting detection reagents (GE Healthcare) according to the manufacturer’s instructions. The band intensities were quantified by densitometric analysis using ImageJ software (National Institutes of Health).

### PGE2 EIA

PGE2 production from cells was determined with an enzyme immunoassay (EIA) kit (Cayman Chemical) according to the manufacturer’s instructions. Briefly, PGE2-acetylcholinesterase conjugate, mouse monoclonal anti-PGE2 antibody, and either standard or sample were added to each well of an EIA plate coated with goat polyclonal anti-mouse IgG. After incubation for 18 h at 4 °C, the plate was washed five times to remove all unbound reagents. Ellman’s reagent was then added to each well, and the developed plate was read at 405 nm with a WALLAC ARVO SX multilabel counter (PerkinElmer, Inc., Waltham, MA, USA).

### Real-time RT-PCR

Total RNA was isolated from A549 cells using a Fast Pure RNA kit (Takara Bio). The first-strand cDNA was synthesized from total RNA with PrimeScript Reverse Transcriptase (Takara Bio). The cDNA was used as a template for real-time PCR analysis: reactions were performed in a Stratagene Mx3000P^®^ QPCR system (Agilent Technologies, La Jolla, CA, USA). The sequences of specific primers for human COX-2 were 5’-GAGAAAACTGCTCAACACCGGA-3’ (sense) and 5’-CACAACGTTCCAAAATCCCTTG-3’ (antisense), those for human COL1A1 were 5’-CACACGTCTCGGTCATGGTA-3’ (sense) and 5’- AAGAGGAAGGCCAAGTCGAG-3’ (antisense). Glyceraldehyde-3-phosphate dehydrogenase (GAPDH) mRNA was determined as a positive control. Each sample was assayed in a 20 µL amplification reaction mixture containing cDNA, primers (5 µM each of sense and antisense primers) and 2x KAPA SYBR^®^ FAST qPCR Master Mix (KAPA Biosystems, Woburn, MA, USA). The amplification program included 40 cycles of 95 °C for 3 sec and 60 °C for 30 sec. Fluorescent products were detected at the last step of each cycle. The obtained values were within the linear range of the standard curve and were normalized to GAPDH mRNA expression.

### Cell cycle analysis

The cell cycle was analyzed by propidium iodide (PI) staining. A549 cells were trypsinized and fixed with 70% ethanol for 60 min on ice. RNA was removed by treatment with RNase A (0.34 mg/mL), then the cells were incubated with 50 mg/mL of PI for 30 min on ice. Stained cells were analyzed using a FACS Calibur (BD Biosciences, San Jose, CA, USA), and ten thousand cells were assessed. The DNA profile indicated the relative abundance of the cell population in G0/G1, S and G2/M phases. The percentages of cells in each phase of the cell cycle were determined by statistical analysis using Cell Quest software (Becton Dickinson, San Jose, CA, USA).

### Proliferation assay

Cell proliferation assays were carried out using the bromodeoxyuridine (BrdU) (colorimetric) cell proliferation ELISA (Roche, Mannheim, Germany) according to the manufacturer’s instructions. Briefly, A549 cells were seeded in 96-well plates and treated with vehicle or reagents in a final volume of 100 µL/well. Subsequently, 10 µL of BrdU-labeling solution was added to each well, and incubation was continued for 2 h. After removal of the BrdU-labeling solution, cells were dried and incubated with the kit’s FixDenat solution for 30 min at room temperature. After denaturation of the DNA, samples were incubated with peroxidase-conjugated anti-BrdU antibody for 90 min. Unbound antibodies were washed off and 100 µL of the luminescence substrate was added. The luminescence was quantified using a WALLAC ARVO SX multilabel counter.

Cell proliferation was also analyzed by counting cells. A549 cells were seeded in 24-well plates and treated with TGF-β1 and PGE2. After incubation, cells were trypsinized and counted using a TC10^TM^ Automated cell counter (Bio-Rad Laboratories).

### Fluorescence imaging

For F-actin staining, cells were fixed with 4% paraformaldehyde for 10 minutes at room temperature, and permeabilized with 0.5% Triton X-100 for 5 min on ice. Fixed cells were incubated with 100 nM rhodamine phalloidin for 30 min at room temperature. Counterstaining with Hoechst 33258 (10 µg/mL) was used to verify the location and integrity of the nuclei. Stained cells were analyzed using a confocal laser scanning microscope (TCS SP2; Leica, Mannheim, Germany) equipped with a HCX PLApo 63 x 1.32 NA oil objective lens. Leica confocal software (TCS SP2, version 2.6.1) was used for image acquisition and processing.

### Cell migration assay

Cell migration was analyzed by using 24-well Transwell plates (6.5 mm diameter; 8 µm pore size polycarbonate membrane, Corning, Lowell, MA, USA). The upper compartment was seeded with A549 cells (2 x 10^4^ cells) in basal culture medium. After 24 h, the medium was replaced with fresh medium containing TGF-β1. The upper chamber contained 5% FBS instead of 10%. After incubation for a further 24 h, cells were fixed with 4% paraformaldehyde for 10 minutes at room temperature, and incubated with 50 µg/mL of PI for 30 minutes at room temperature. Non-migrated cells on the upper surface of the membrane were removed and cells that had migrated through the membrane to the lower surface were counted by using a fluorescence microscope (BZ-9000; KEYENCE).

### Statistics

Results are expressed as mean ± SE. The statistical significance of differences between control and other groups was calculated by using Dunnett’s test or unpaired t test with the Instat version 3.0 statistical package (GraphPad Software, San Diego, CA, USA). The criterion of significance was set at P < 0.05.

## Results

### TGF-β1 downregulates COX-2 expression and PGE2 production in A549 cells

**Figure 1:**
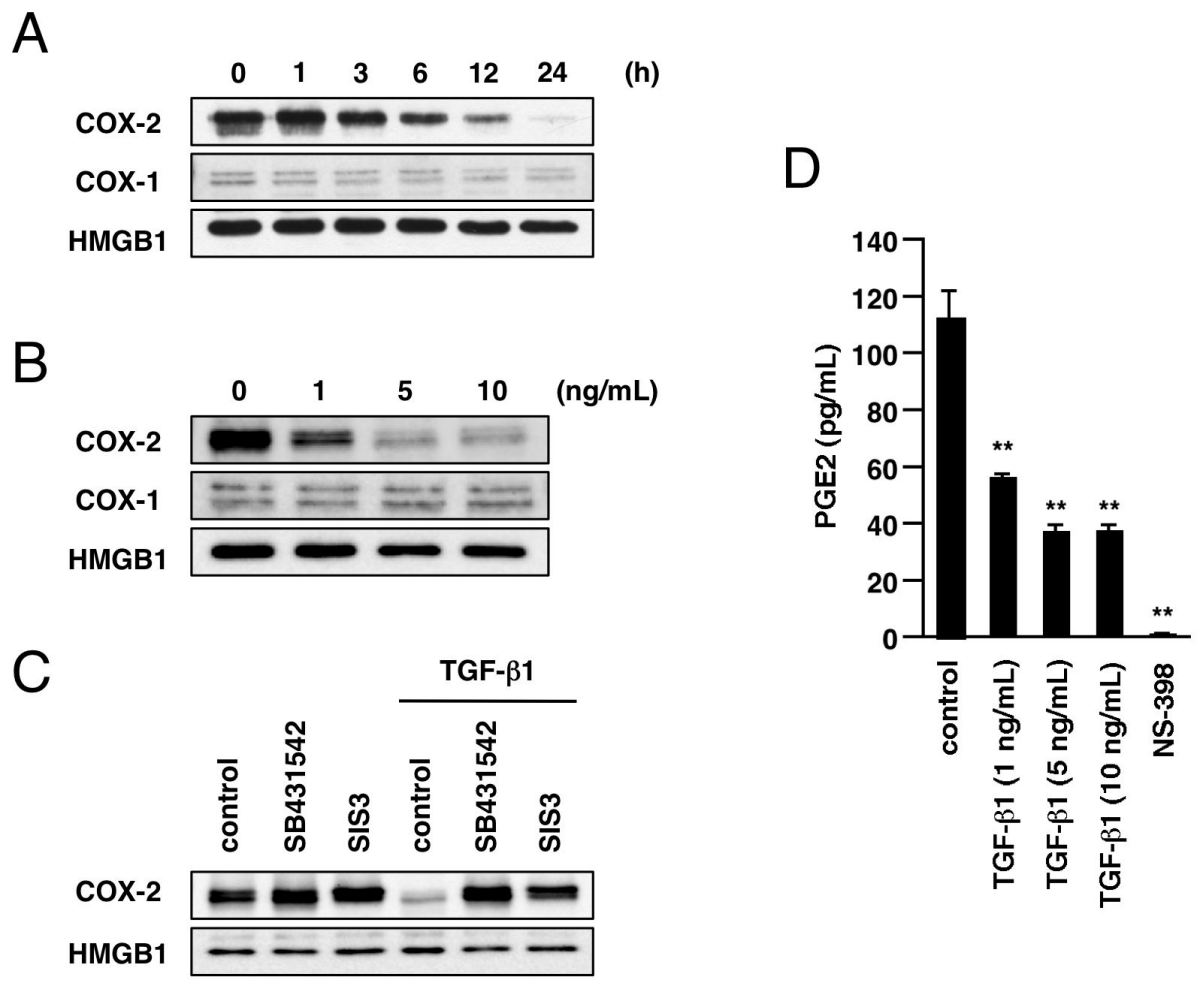
Downregulation of COX-2 expression and PGE2 by TGF-β1 in A549 cells. (A) Expression of COX-2 protein was detected by immunoblotting as described in Materials and Methods. Treatment with TGF-β1 (5 ng/mL) suppressed expression of COX-2 but not COX-1 in A549 cells. HMGB1 was measured as a loading control. (B) Dose-dependent downregulation of COX-2 by TGF-β1. Twenty-four hours after TGF-β1 stimulation (1-10 ng/mL), expression of COX-2 and COX-1 was detected. (C) SB431542 (10 µM) or SIS3 (30 µM) blocked TGF-β1-induced COX-2 downregulation. Cells were pretreated for 60 min with the inhibitor, and then incubated with or without TGF-β1 (5 ng/mL) for 24 h. (D) TGF-β1 suppressed COX-2 production in A549 cells. Cells were treated with TGF-β1 (1-10 ng/mL) or NS-398 (50 µM) for 24 h, then the concentration of PGE2 in culture medium was measured by EIA as described in Materials and Methods. Each value represents the mean ± SE (n = 4). Significant differences between the indicated groups and control group are indicated by **(P < 0.01).

In many types of cancer, TGF-β1 is secreted into the tumor microenvironment and acts as a tumor promoter. To investigate TGF-β1-induced progression of human lung cancer, we stimulated human lung cancer A549 cells with TGF-β1. COX-2 is constitutively overexpressed in A549 cells under standard cell culture conditions. Surprisingly, we found that the expression of COX-2 protein was dramatically diminished by treatment with 5 ng/mL of TGF-β1 (Figure 1A). The decrease of COX-2 protein at 24 h after stimulation was TGF-β1 dose-dependent (Figure 1B). On the other hand, the expression of COX-1 in A549 cells was low, and was not changed by TGF-β1 treatment (Figure 1A, B). TGF-β1 activates TGF-β serine/threonine kinase receptors. Following ligand binding, the TGF-β type I receptor (TßRI) and type II receptor (TßRII) form a tetrameric receptor heterocomplex that lead to phosphorylation of Smad proteins, the main downstream effector molecules for these receptors, by the kinase activities of the cytoplasmic domain of TßRI. Treatment with TßRI inhibitor SB431542 or Smad3 inhibitor SIS3 [27] blocked COX-2 downregulation by TGF-β1 (Figure 1C). The results indicate that TGF-β1-induced downregulation of COX-2 is associated with activation of TGF-β receptors and the Smad signaling pathway.

The COX enzymes convert arachidonic acid into prostaglandin H2, which is further processed into various prostaglandins, including PGE2, the predominant prostaglandin in lung [28,29]. To evaluate PGE2 production in A549 cells, we measured the extracellular concentration of PGE2 in culture medium using enzyme immunoassay. As shown in Figure 1D, extracellular concentration of PGE2 was decreased by treatment with TGF-β1 for 24 h. A selective inhibitor of COX-2, NS-398, completely eliminated PGE2 from the culture medium, indicating that production of PGE2 in A549 cells mainly depends on the function of COX-2. These results show that TGF-β1 stimulation strongly suppresses the expression of COX-2 and results in a reduction of PGE2 production in A549 cells.

### Suppression of COX-2 expression by TGF-β1 is mediated by transcriptional repression

**Figure 2:**
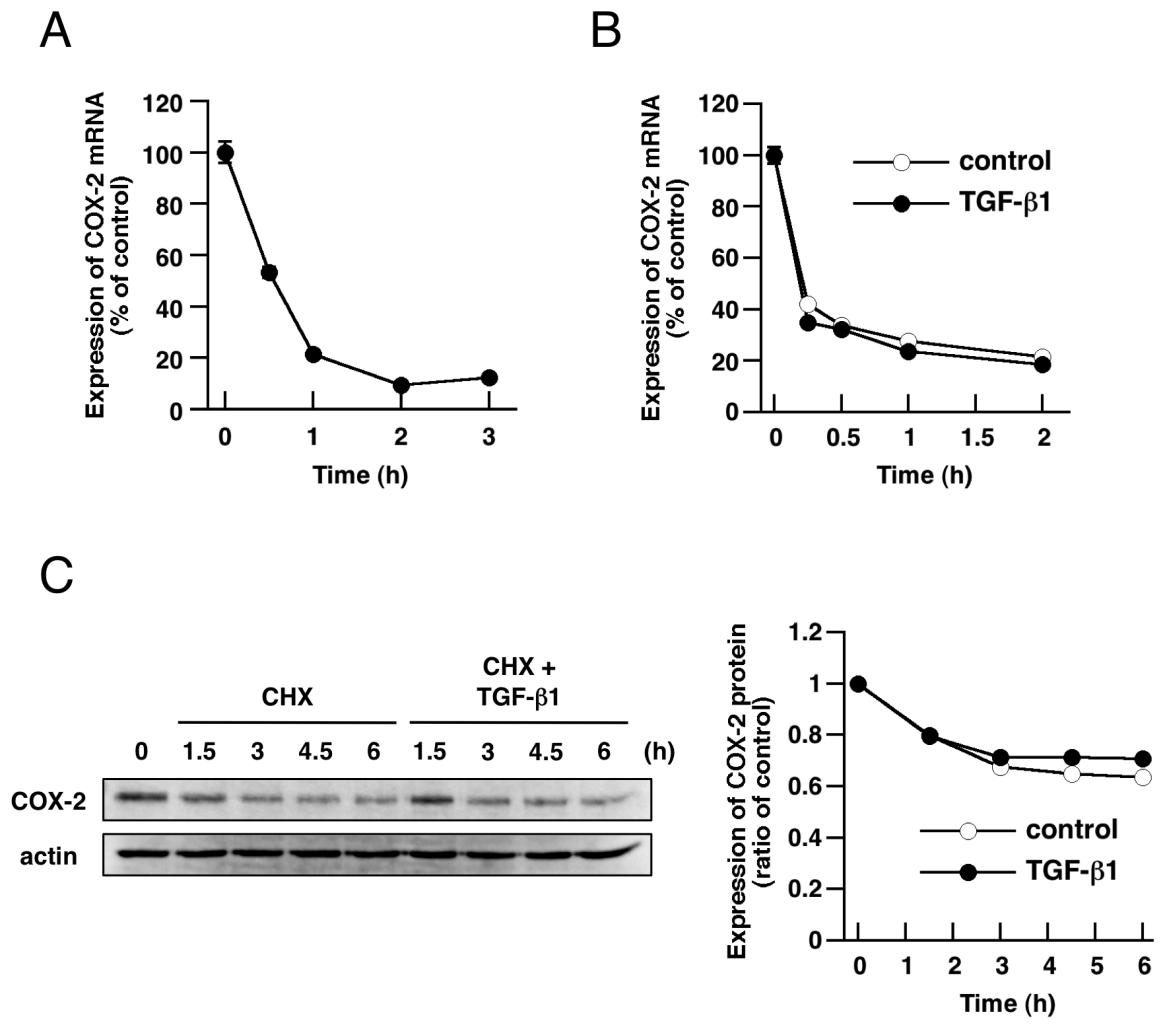
TGF-β1-induced suppression of COX-2 expression by transcriptional repression. (A) Time-dependent decrease of COX-2 mRNA expression by treatment with TGF-β1 (5 ng/mL). COX-2 mRNA level was measured by real-time RT-PCR as described in Materials and Methods. (B) Cells were pretreated with actinomycin D (5 µg/mL) for 1 h and incubated with or without TGF-β1 (5 ng/mL) for the indicated times. TGF-β1 did not affect decrease of COX-2 mRNA by actinomycin D. COX-2 expression levels were normalized for GAPDH expression levels and expressed as a percentage of that in non-treated cells (control). Each value represents the mean ± SE (n = 3). (C) Cells were incubated with CHX (50 µg/mL) or CHX plus TGF-β1 (5 ng/mL) for the indicated times and COX-2 protein expression was detected by immunoblotting. TGF-β1 did not affect decrease of COX-2 protein by CHX. The band intensities were quantified and COX-2/actin values were expressed as relative to those of control. Each value represents the mean ± SE (n = 4).

We next investigated the mechanism of TGF-β1-induced COX-2 downregulation in A549 cells. COX-2 protein expression could be affected by protein degradation, altered mRNA stability and altered transcription rate. Using real-time RT-PCR analysis, we examined the expression of COX-2 mRNA upon TGF-β1 treatment from 0.5 to 3 h. As shown in Figure 2A, COX-2 mRNA was decreased rapidly, reaching approximately 50% of the control within 30 min after stimulation. This result indicates that the decrease of COX-2 protein after TGF-β1 stimulation is mainly the result of a decrease of COX-2 mRNA. To examine whether TGF-β1 affects COX-2 mRNA stability, cells were pretreated with the transcriptional inhibitor actinomycin D before TGF-β1 stimulation. Then, COX-2 mRNA level was examined by real-time RT-PCR. Control cells treated with actinomycin D alone showed a significant decrease in COX-2 mRNA. Treatment with TGF-β1 did not affect COX-2 mRNA stability, as the degradation rate was identical to that observed in control cells (Figure 2B). Furthermore, we examined COX-2 protein stability using the protein synthesis inhibitor cycloheximide (CHX). Cells were treated with CHX or CHX plus TGF-β1, and then COX-2 protein level was examined by immunoblotting. As shown in Figure 2C, COX-2 protein was decreased in control cells treated with CHX alone and TGF-β1 did not affect COX-2 protein stability. These results indicate that TGF-β1 does not affect COX-2 protein degradation and mRNA stability, but rather acts at the transcriptional level.

### Downregulation of COX-2 is related to TGF-β1-induced growth inhibition of A549 cells

**Figure 3:**
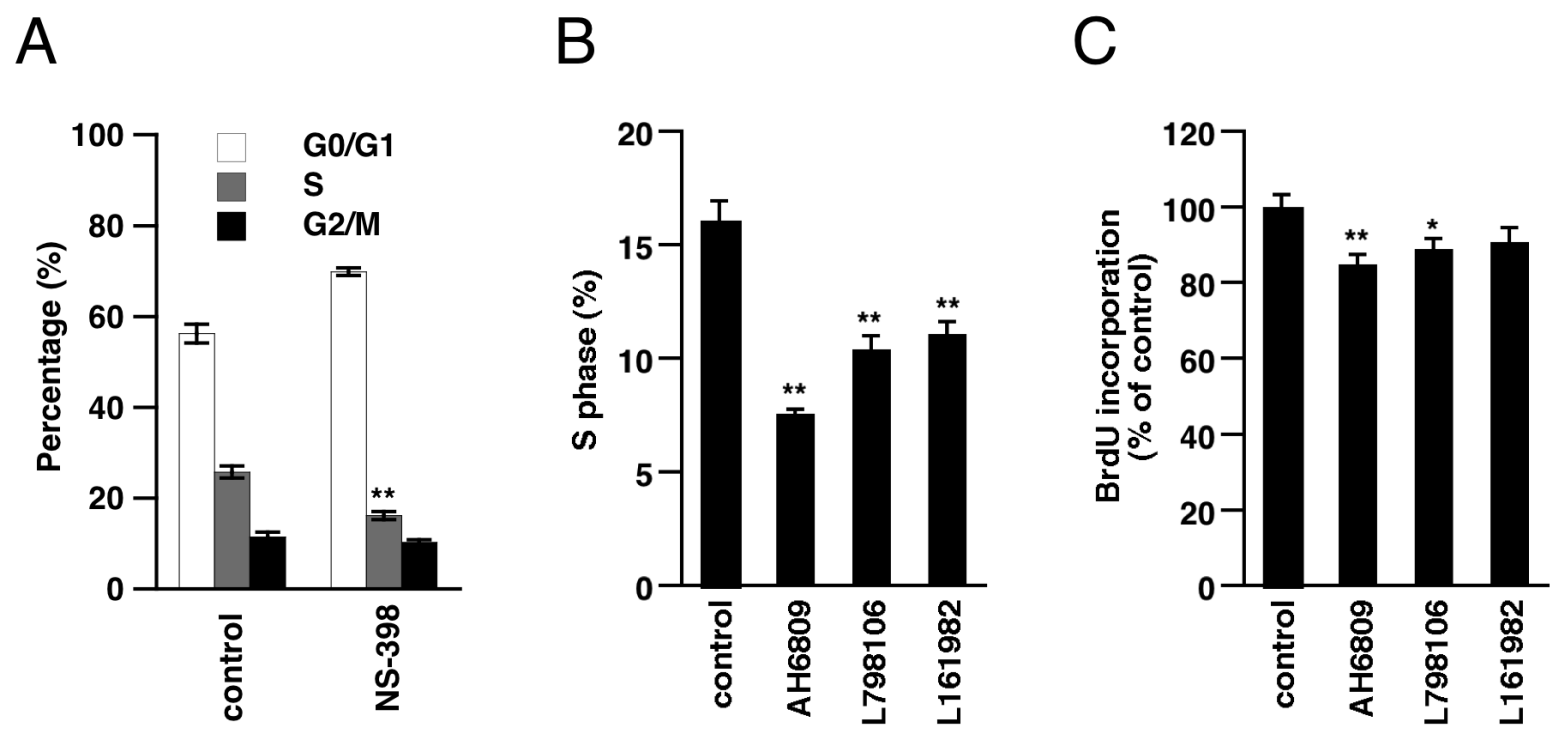
Involvement of COX-2/PGE2 signaling in proliferation of A549 cells. (A) A549 cells were incubated with NS-398 (50 µM) for 3 days and the cell cycle was analyzed as described in Materials and Methods. Treatment with NS-398 decreased the ratio of S phase cells. Each value represents the mean ± SE (n = 3). (B) Cells were treated with AH6809 (30 µM), L798106 (30 µM) or L161982 (30 µM) for 3 days, then the cell cycle was analyzed and the percentage of S phase was calculated. Each value represents the mean ± SE (n = 5-7) (C) Treatment with AH6809 (30 µM), L798106 (30 µM) or L161982 (30 µM) for 3 days suppressed proliferation of A549 cells. Cell proliferation was examined by BrdU assay as described in Materials and Methods. BrdU incorporation levels were expressed relative to that in non-treated cells (control). Each value represents the mean ± SE (n = 6). Significant differences between the indicated groups and control group are indicated by *(P < 0.05) and **(P < 0.01).

To clarify the significance of TGF-β1-induced COX-2 downregulation, we next investigated what roles COX-2 and PGE2 play in the biological functions of A549 cells. PGE2 activates EP receptors, which are classified into 4 subtypes; Gq protein-coupled EP1, Gi protein-coupled EP3, Gs protein-coupled EP2 and EP4. These EP receptors have been implicated in proliferation of various cancer cells [22,30-32]. It was also suggested that PGE2 and EP receptors are involved in proliferation of NSCLC cells [33]. We examined the involvement of COX-2/PGE2 in the cell cycle of A549 cells. Treatment with COX-2 inhibitor NS-398 for 3 days resulted in a decrease of S phase and an increase of G0/G1 phase (Figure 3A). As shown in Figure 3B, EP1/2 antagonist AH6809, EP3 antagonist L798106 or EP4 antagonist L161982 also significantly decreased the ratio of S phase cells. We further examined cell proliferation by using BrdU assay; incorporation of BrdU indicates DNA synthesis during cell proliferation. Treatment with these EP antagonists suppressed BrdU incorporation in A549 cells (Figure 3C). We also analyzed the expression of EP receptors in A549 cells by RT-PCR, and confirmed expression of all 4 receptor subtypes (data not shown). These results indicate that constitutive production of PGE2, at least to some extent, contributes to proliferation of A549 cells via EP receptors under standard culture conditions.

**Figure 4:**
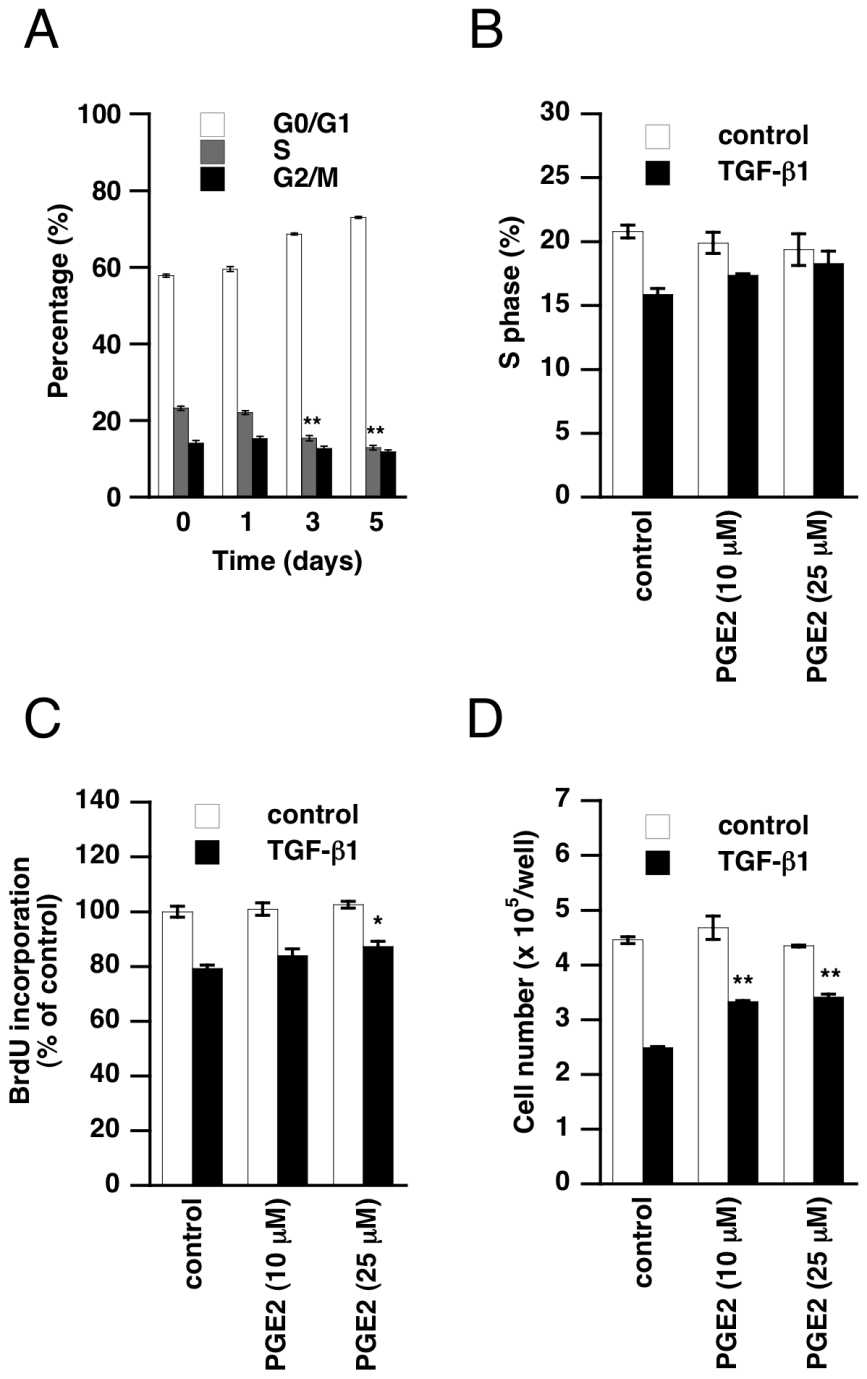
Abrogation of TGF-β1-induced growth inhibition by PGE2. (A) A549 cells were treated with TGF-β1 (5 ng/mL) for the indicated times and the cell cycle was analyzed. Treatment with TGF-β1 decreased the ratio of S phase cells. (B-D) Twenty-four hours after incubation with or without TGF-β1 (5 ng/mL), cells were supplemented with PGE2 (10, 25 µM) and further incubated for 2 days. Proliferation was examined by cell cycle analysis (B), BrdU assay (C) and cell counting (D) as described in Materials and Methods. PGE2 abrogated TGF-β1-induced growth inhibition. Each value represents the mean ± SE (n = 3-5). Significant differences between the indicated groups and control group are indicated by *(P < 0.05) and **(P < 0.01).

Therefore, TGF-β1-induced downregulation of COX-2 could affect proliferation of A549 cells. Stimulation with TGF-β1 also decreased S phase and increased G0/G1 phase of A549 cells (Figure 4A). This cell cycle arrest was observed from 3 days after treatment, when A549 cells might be expressing less COX-2. Indeed, TGF-β1-induced reduction of S phase cells tended to be abrogated by exogenous PGE2 in a dose-dependent manner (Figure 4B). To further investigate the effect of TGF-β1 on proliferation of A549 cells, we measured cell proliferation by means of BrdU assay. We confirmed that BrdU incorporation was decreased by TGF-β1 stimulation for 3 days, and this TGF-β1-induced suppression of BrdU incorporation was partly attenuated in the presence of PGE2 (Figure 4C). We also found that TGF-β1 did not affect the expression pattern of EP receptor subtypes in A549 cells (data not shown). These results support the idea that TGF-β1-induced COX-2 downregulation is associated with suppression of proliferation of A549 cells. In fact, the cell count in samples treated with TGF-β1 for 3 days was decreased, compared with the case of non-treated cells, and addition of PGE2 increased the cell count of TGF-β1-treated cells (Figure 4D). Thus, our results suggest that TGF-β1-induced growth inhibition of A549 cells is partly mediated by downregulation of COX-2. Since cell growth arrest at G0/1 phase is essential for cell differentiation, TGF-β1-induced growth inhibition of A549 cells might be associated with EMT induced by TGF-β1 in A549 cells.

### Downregulation of COX-2 accelerates TGF-β1-induced fibrotic EMT response

**Figure 5:**
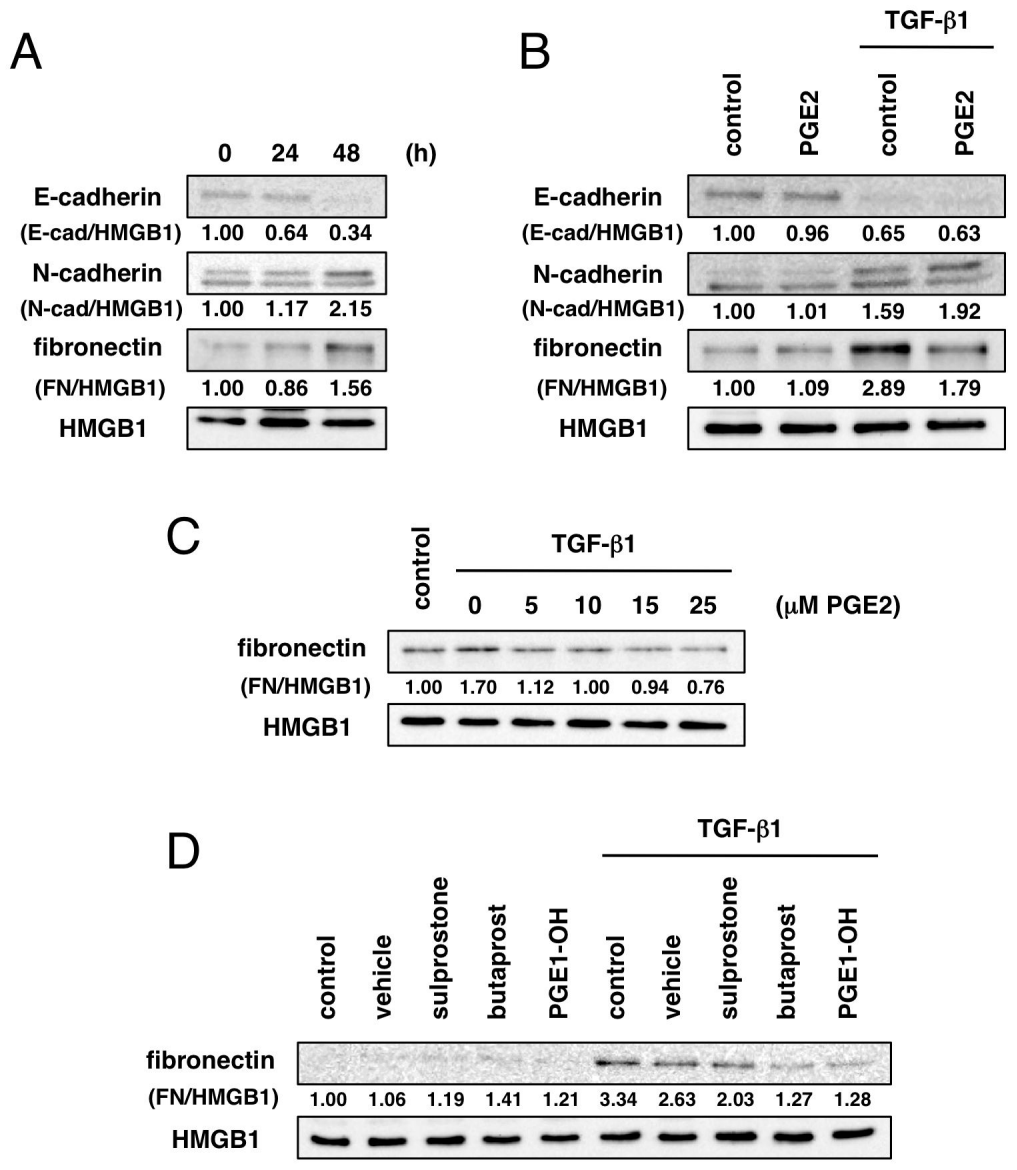
Suppressive effect of EP receptor agonists on TGF-β1-induced fibronectin expression. (A) A549 cells were treated with TGF-β1 (5 ng/mL) for the indicated times and expression of E-cadherin, N-cadherin and fibronectin was detected by immunoblotting. (B) Forty-eight hours after incubation with TGF-β1 (5 ng/mL) and PGE2 (25 µM), the expression of E-cadherin, N-cadherin and fibronectin was evaluated. (C) Cells were incubated with TGF-β1 (5 ng/mL) and PGE2 (5-25 µM) for 48 h and the expression of fibronectin was examined. PGE2 suppressed fibronectin induction by TGF-β1. (D) Cells were treated with TGF-β1 (5 ng/mL) and vehicle (methyl acetate), sulprostone, butaprost or PGE1-OH (20 µM) for 48 h and expression of fibronectin was evaluated. Treatment with butaprost or PGE1-OH inhibited TGF-β1-induced increase of fibronectin expression. HMGB1 was detected as a loading control. The band intensities were quantified by densitometry and expressed as relative to those of each control.

TGF-β1 is well known to be a potent inducer of EMT, which is a phenotype change that involves differentiation of epithelial cells into fibroblastoid-like cells. In the process of EMT, epithelial marker proteins, such as E-cadherin, are lost and mesenchymal proteins, including N-cadherin and fibronectin, are upregulated. We confirmed the decrease of E-cadherin and increase of N-cadherin at 48 h after TGF-β1 stimulation. At the same time, expression of fibronectin was elevated (Figure 5A). To investigate the involvement of COX-2 downregulation in these TGF-β1-induced EMT responses, cells were costimulated with TGF-β1 and PGE2. As shown in Figure 5B, PGE2 (25 µM) did not affect the loss of E-cadherin or upregulation of N-cadherin induced by TGF-β1. On the other hand, the TGF-β1-induced upregulation of fibronectin was markedly inhibited by exogenous PGE2. PGE2 suppressed TGF-β1-induced fibronectin expression at doses as low as 5-10 µM (Figure 5C). Next, we also examined the effect of EP receptor agonists on fibronectin expression. Treatment with EP2 receptor agonist butaprost or EP4 receptor agonist PGE1-OH inhibited the increase of fibronectin induced by TGF-β1, whereas EP1/3 receptor agonist sulprostone had no effect (Figure 5D). These results suggest that EP2 and EP4 receptors mediate the suppressive effect of PGE2 on TGF-β1-induced fibronectin expression.

**Figure 6:**
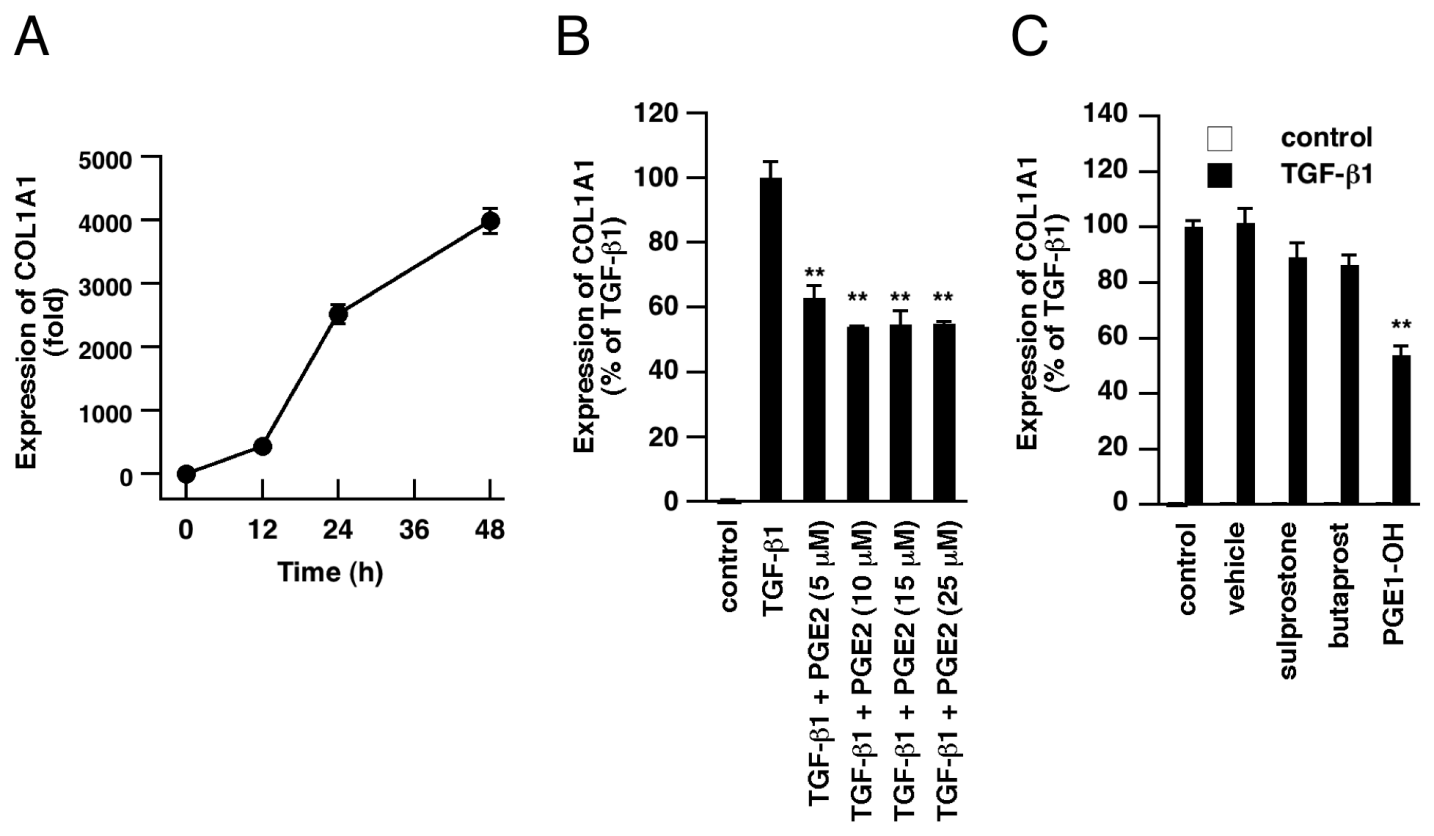
Inhibition of TGF-β1-induced collagen I gene expression by EP receptor agonists. (A) Cells were stimulated with TGF-β1 (5 ng/mL) for the indicated times; then total RNA was extracted and COL1A1 gene expression was examined by real-time RT-PCR. (B) Twenty-four hours after treatment with TGF-β1 (5 ng/mL) and PGE2 (5-25 µM), COL1A1 gene expression was examined. PGE2 abrogated TGF-β1-induced COL1A1 gene expression. (C) Cells were treated with TGF-β1 (5 ng/mL) and vehicle (methyl acetate), sulprostone, butaprost or PGE1-OH (20 µM) for 24 h, and the COL1A1 mRNA level was measured. PGE1-OH suppressed COL1A1 induction by TGF-β1. COL1A1 gene expression levels were normalized for GAPDH expression and are shown as a percentage of that in TGF-β1-treated cells. Each value represents the mean ± SE (n = 3). Significant differences between the indicated groups and control group are indicated by **(P < 0.01).

Fibronectin is a component of ECM and altered fibronectin expression is associated with fibrotic disorders. We further investigated whether TGF-β1 induces expression of collagen I, which is a major ECM component in fibrotic tissues [34-36]. The COL1A1 (encoding collagen I) transcript level was elevated by stimulation with TGF-β1 (5 ng/mL) in a time-dependent manner (Figure 6A). This TGF-β1-induced expression of COL1A1 was attenuated by supplementation with exogenous PGE2 (Figure 6B). As well as PGE2, PGE1-OH strongly suppressed COL1A1 induction by TGF-β1, whereas butaprost and sulprostone exhibited only modest suppressive effects (Figure 6C). Together, these results demonstrate that downregulation of COX-2 and PGE2 production by TGF-β1 is involved in ECM component production in A549 cells.

### TGF-β1-induced suppression of PGE2 production is involved in actin remodeling in A549 cells

**Figure 7:**
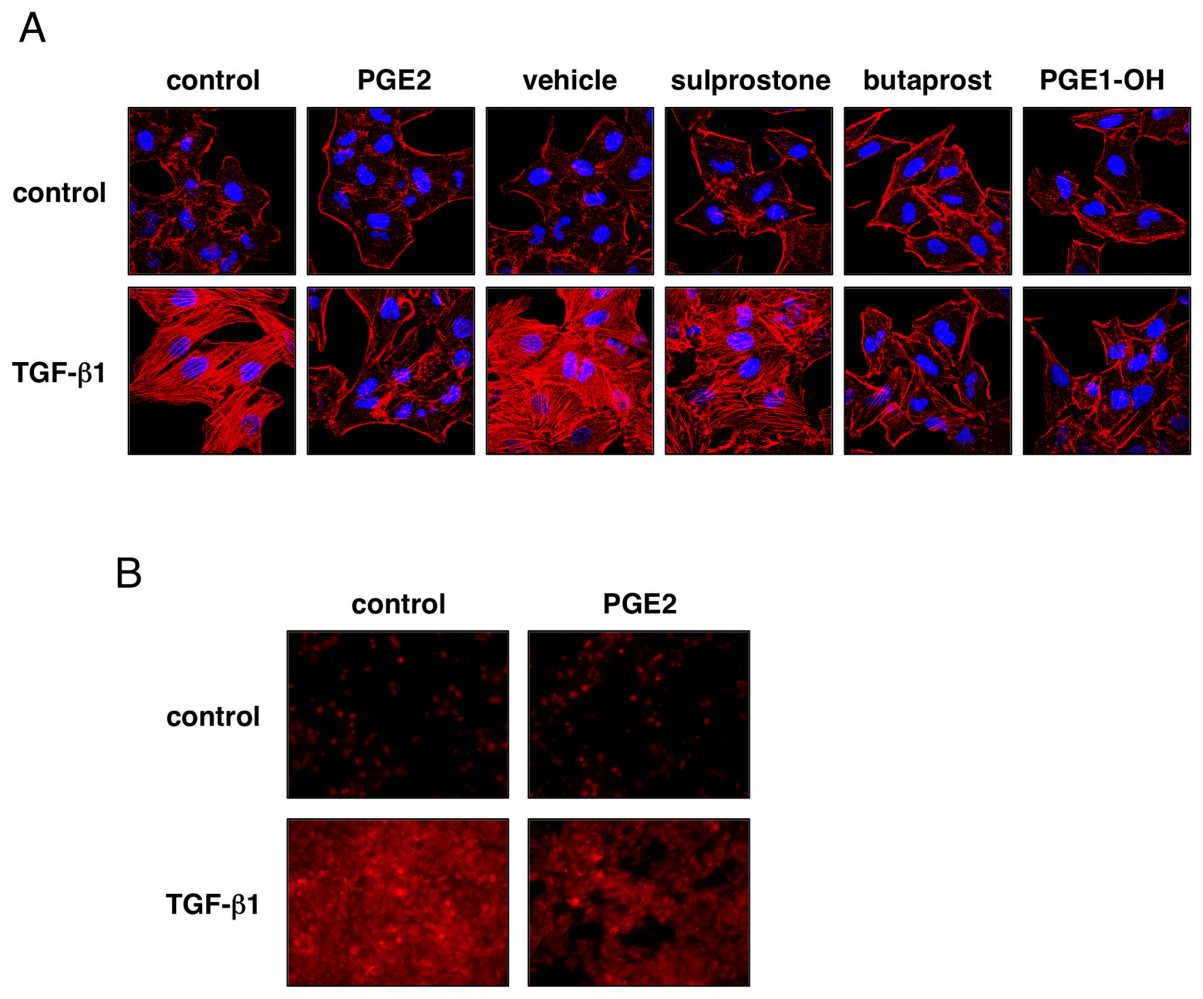
Suppressive effect of EP agonists on TGF-β1-induced actin stress fiber formation and migration. (A) A549 cells were incubated with TGF-β1 (5 ng/mL) and PGE2 (10 µM), vehicle (methyl acetate), sulprostone, butaprost or PGE1-OH (20 µM) for 12 h. Then F-actin was stained using rhodamine phalloidin (red) and stained cells were analyzed using a confocal laser-scanning microscope at x63 magnification. TGF-β1-induced actin stress fiber formation was suppressed by PGE2, butaprost or PGE1-OH. To verify the location of nuclei, cells were stained with Hoechst33258 (blue). (B) Cell migration was examined by Transwell assay as described in Materials and Methods. A549 cells were treated with TGF-β1 (5 ng/mL) and PGE2 (10 µM) for 24 h; then cells were stained and the lower membrane surfaces were photographed through a fluorescence microscope at x20 magnification. Migrated cells were decreased in the presence of PGE2.

We also investigated actin stress fiber formation, which has been considered as a hallmark of fibroblastoid cells, as well as a part of EMT. As shown in Figure 7A, TGF-β1 (5 ng/mL) strikingly induced actin polymerization, whereas actin stress fibers were hardly detected in PGE2-treated cells. Treatment with butaprost or PGE1-OH also inhibited TGF-β1-induced actin stress fiber formation. On the other hand, sulprostone slightly suppressed actin polymerization by TGF-β1. Since actin stress fibers are related to cell motility, we also examined cell migration by Transwell assay. As shown in Figure 7B, stimulation with TGF-β1 increased cell migration within 24 h, whereas the number of migrated cells was decreased in the presence of PGE2. These results suggest that TGF-β1-induced suppression of PGE2 production is related to actin stress fiber formation and migration of A549 cells.

## Discussion

In this study, we found that TGF-β1 induces COX-2 downregulation in human NSCLC A549 cells, and we investigated its physiological significance. Our results reveal a novel mechanism, in which TGF-β1-induced fibrotic responses are mediated by suppression of COX-2 expression in human lung cancer cells.

First, we found that TGF-β1 stimulation drastically suppressed COX-2 expression in A549 cells. This change was accompanied by a decrease in production of PGE2, which is the predominant PG in lung [28,29]. Considering that COX-1 expression was not affected by TGF-β1, the suppression of PGE2 production could be due to COX-2 downregulation. TßRI inhibitor and specific inhibitor of Smad3 blocked TGF-β1-induced COX-2 downregulation, suggesting involvement of the TGF/Smad signaling pathway. We also found that TGF-β1 stimulation immediately decreased COX-2 mRNA in A549 cells. When transcription was blocked by actinomycin D treatment, TGF-β1 had no effect on COX-2 mRNA, suggesting that the decrease of COX-2 mRNA by TGF-β1 is due to transcriptional repression, not by decrease of mRNA stability. Various transcription factors, including nuclear factor-κB (NF-κB), activator protein-1 (AP-1) and STAT3/5, are known to regulate COX-2 gene transcription [37-39]. We examined which transcription signals are involved in COX-2 gene expression in A549 cells. However, since inhibitors of NF-κB, STAT3, STAT5, or p38, JNK, ERK MAPK pathways (upstream of NF-κB or AP-1) did not decrease COX-2 expression in A549 cells (data not shown), these pathways might not predominantly contribute to the constitutive induction of COX-2 in A549 cells. Nevertheless, in mammary epithelial cells, it has been reported that ligand-induced activation of Smad signaling leads to inhibition of STAT5-mediated transcription by blocking STAT5 association with the co-activator CREB-binding protein (CBP) [40]. Although further investigation may be necessary in order to clarify the mechanism of TGF-β1-induced downregulation of COX-2 in A549 cells, it is possible that Smad signaling antagonizes transcriptional factor function, such as STAT5 association with CBP, similarly to the case in mammary epithelial cells.

Next, we investigated the significance of COX-2 downregulation in TGF-β1-induced biological events. We found that COX-2 and EP receptors are involved in the proliferation of A549 cells. In addition, growth inhibition by TGF-β1 was partly rescued by exogenous PGE2. These results suggest that TGF-β1 halts the proliferation of A549 cells by downregulating COX-2, at least to some extent. The cell growth arrest is generally associated with cell differentiation. Indeed, TGF-β1-treated A549 cells underwent EMT, showing a decrease of E-cadherin and increases of N-cadherin, fibronectin and collagen I. Though cadherin switching was not affected, induction of fibronectin and collagen I expression was suppressed by exogenous PGE2. Fibronectin and collagen are extracellular matrix components, and excess deposition of those proteins directly contributes to fibrotic disorders. Recently, it has been suggested that EMT-derived fibroblast-like cells play an important role in the development of fibrosis [12,13]. Our results suggest that TGF-β1-induced EMT is also involved in promotion of fibrosis in lung cancer tissues. Furthermore, we found that suppression of PGE2 synthesis is important for this TGF-β1-induced fibrotic response in A549 cells. Exogenous PGE2 is known to inhibit collagen and α-smooth muscle actin expression in fibroblasts [41-43]. This is the first report to demonstrate TGF-β1-induced downregulation of COX-2 and its contribution to fibrotic response in A549 cells. In addition, we found that exogenous PGE2 suppressed TGF-β1-induced actin stress fiber formation and migration. Based on the effects of EP receptor agonists, EP4 receptor could mediate the suppressive effect of PGE2 on actin polymerization. Actin stress fibers are not only a hallmark of proto-fibroblasts, but have also been recognized as an important contributor to cell migration. PGE2 has been reported to inhibit migration or chemotaxis of fibroblasts via EP2 or EP4 receptors, which increase cAMP [44-46]. Considering the effect of butaprost and PGE1-OH, the suppressive effect of PGE2 on TGF-β1-induced fibrotic EMT may be mediated through EP2 and EP4, especially the EP4 receptor. Although it has been reported that COX-2 and PGE2 promote migration in many types of cancer [47-49], our results suggest that PGE2 rather exerts an inhibitory effect on TGF-β1-induced migration of A549 cells.

Our study identifies suppression of COX-2 expression and PGE2 synthesis as important elements in the TGF-β1-induced fibrotic response in A549 cells. Since PGE2 produced by lung cancer cells would affect not only cancer cells themselves, but also bystander cells, COX-2 downregulation by TGF-β1 might result in proliferation and activation of tumor-associated fibroblasts. It has been suggested that the degree of collagenization in the fibrotic focus in lung tumors is closely correlated with tumor progression and prognosis [9]. TGF-β1 is secreted in large amounts from cancer cells, and could induce fibrogenesis in tumor tissues. Therefore, inhibition of TGF-β1-induced COX-2 downregulation or activation of EP receptors might be beneficial for treatment of fibrosis in lung cancer tissues or metastasis.

Since COX-2 is overexpressed in many lung cancers and COX-2/PGE2 signaling has been implicated in cancer progression, it is considered that use of COX-2 inhibitors is beneficial as lung cancer therapy. Our results suggest that PGE2 rather has inhibitory effect on TGF-β1-induced fibrotic response in A549 cells. Thus, inhibition of COX-2 could promote fibrotic processes in tumor tissues. Indeed, treatment with COX-2 inhibitor NS-398 facilitated expression of fibronectin induced by 1 ng/mL of TGF-β1 (Figure S1). This result supports that inhibition of COX-2 accelerates TGF-β1-induced fibronectin expression in A549 cells. It has also been reported that COX-2 inhibitor induces EMT in human lung cancer cells [50]. Considering these findings, it is possible that applying COX-2 inhibitors have negative effects on lung cancer treatment, such as induction of fibrosis or metastasis.

Expression of COX-2 is involved in synthesis of not only PGE2, but also other prostanoids, such as PGD2. PGD2 and its metabolite 15-deoxy-D^12,14^ PGJ2 (15d-PGJ2) have been reported to induce apoptosis in A549 cells, human cervical cancer cells and human leukemia cells [51,52]. PGD2 also inhibits TGF-β1-induced EMT in MDCK cells, and 15d-PGJ2 decreases the metastatic potential of breast cancer cells [53,54]. Therefore, TGF-β1-induced COX-2 suppression might be expected to promote cancer progression.

In conclusion, we have shown here that TGF-β1 stimulation suppresses COX-2 expression at the transcriptional level and results in decreased PGE2 production in A549 cells. The COX-2 suppression appears to be involved in the production of ECM components and actin remodeling induced by TGF-β1. Our results indicate that blockade of PGE2 signaling in A549 cells is required for an effective fibrotic response to TGF-β1. PGE2 receptors could be a novel target for treatment of certain lung cancer metastasis and tumor fibrosis.

## Supporting Information

Figure S1 **Acceleration of TGF-β1-induced fibronectin expression by COX-2 inhibitor.** A549 cells were treated with TGF-β1 (1 ng/mL) and NS-398 (50 mM) for 48 h and the expression of fibronectin was detected by immunoblotting. HMGB1 was detected as a loading control. The band intensities were quantified by densitometry and expressed as relative to those of control.
(PDF)
