# Supplementary material for: TGF-β1 Downregulates COX-2 Expression Leading to Decrease of PGE2 Production in Human Lung Cancer A549 Cells, Which Is Involved in Fibrotic Response to TGF-β1
Source: PLoS One. 2013 Oct 2;8(10):e76346. doi: 10.1371/journal.pone.0076346 (PMC3788736; doi:10.1371/journal.pone.0076346)
Supplement: Figure S1 — Acceleration of TGF-β1-induced fibronectin expression by COX-2 inhibitor. A549 cells were treated with TGF-β1 (1 ng/mL) and NS-398 (50 mM) for 48 h and the expression of fibronectin was detected by immunoblotting. HMGB1 was detected as a loading control. The band intensities were quantified by densitometry and expressed as relative to those of control. (PDF) [file pone.0076346.s001.pdf]

Supporting Information

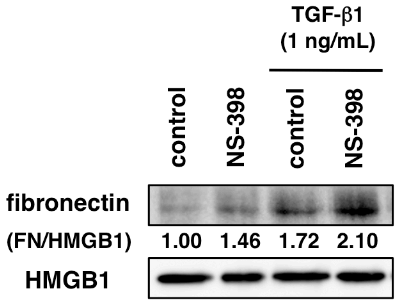

Figure S1 Acceleration of TGF-β1-induced fibronectin expression by COX-2 inhibitor.

A549 cells were treated with TGF-β1 (1 ng/mL) and NS-398 (50 mM) for 48 h and the expression of fibronectin was detected by immunoblotting. HMGB1 was detected as a loading control. The band intensities were quantified by densitometry and expressed as relative to those of control.
